# Integrin β1 in Pancreatic Cancer: Expressions, Functions, and Clinical Implications

**DOI:** 10.3390/cancers14143377

**Published:** 2022-07-11

**Authors:** Jiajia Li, Liyao Peng, Qun Chen, Ziping Ye, Tiantian Zhao, Sicong Hou, Jianguo Gu, Qinglei Hang

**Affiliations:** 1 Department of Gastroenterology, The Affiliated Hospital of Yangzhou University, Yangzhou 225009, China; nylijiajia@126.com (J.L.); shou@yzu.edu.cn (S.H.); 2 Nanjing Drum Tower Hospital, The Affiliated Hospital of Nanjing University Medical School, Nanjing 210000, China; liyaopengnjmu@163.com; 3 Pancreas Center, The First Affiliated Hospital of Nanjing Medical University, Nanjing 210000, China; chenqun9408@163.com; 4 Department of Gastroenterology, The First Affiliated Hospital of Nanjing Medical University, Nanjing 210029, China; yzp1998@126.com; 5 Department of Clinical Medicine, Medical College, Yangzhou University, Yangzhou 225001, China; yzuztt88@163.com; 6 Division of Regulatory Glycobiology, Institute of Molecular Biomembrane and Glycobiology, Tohoku Medical and Pharmaceutical University, Sendai 81-8558, Japan; 7 Department of Experimental Radiation Oncology, The University of Texas MD Anderson Cancer Center, Houston, TX 77030, USA

**Keywords:** integrin β1, pancreatic cancer, signaling pathways, targeted drugs

## Abstract

**Simple Summary:**

Pancreatic cancer (PC) is a highly aggressive malignant tumor with an extremely poor prognosis. Early diagnosis and treatment are key to improving the survival rate of PC patients. Emerging studies show that integrins might contribute to the pathogenesis of PC. This review presents the various signaling pathways that are mediated by integrins in PC and emphasizes the multiple functions of integrin β1 in malignant behaviors of PC. It also discusses the clinical significance of integrin β1 as well as integrin β1-based therapy in PC patients.

**Abstract:**

Pancreatic cancer (PC) is characterized by rapid progression and a high mortality rate. The current treatment is still based on surgical treatment, supplemented by radiotherapy and chemotherapy, and new methods of combining immune and molecular biological treatments are being explored. Despite this, the survival rate of PC patients is still very disappointing. Therefore, clarifying the molecular mechanism of PC pathogenesis and developing precisely targeted drugs are key to improving PC prognosis. As the most common β subunit of the integrin family, integrin β1 has been proved to be closely related to the vascular invasion, distant metastasis, and survival of PC patients, and treatment targeting integrin β1 in PC has gained initial success in animal models. In this review, we summarize the various signaling pathways by which integrins are involved in PC, focusing on the roles of integrin β1 in the malignant behaviors of PC. Additionally, recent studies regarding the feasibility of integrin β1 as a diagnostic and prognostic biomarker in PC are also discussed. Finally, we present the progress of several integrin β1-based clinical trials to highlight the potential of integrin β1 as a target for personalized therapy in PC.

## 1. Introduction

Pancreatic cancer (PC) is the fourth leading cause of cancer-related death worldwide and is characterized by rapid progression, high invasiveness, and resistance to chemotherapeutic agents [1,2]. More than 80% of the PC lesions have invaded surrounding lymph nodes and might even develop distant metastasis [3]. In theory, the possibility of surgical resection in these patients has been lost and only chemotherapy and palliative care are possible. Due to the complex anatomical position of the pancreas, PC surgery is often more complex than other tumors and cannot completely remove the lesions [4]. Additionally, even if the patients undergo surgical treatment, the postoperative recurrence rate is high, and the median survival time after radical surgery is only 18 months [5]. Despite advances in PC diagnosis and combination therapy of PC over the years, the overall 5-year survival rate remains below 5%, and the median survival time is only close to 6 months [6,7]. Therefore, targeting the invasion and metastasis of tumor cells is a cutting-edge research area and an urgent issue in PC.

Tumor cells are endowed with the following characteristics: sustained activation of intracellular proliferation signals, decreased effects of tumor suppressors, resistance to cell apoptosis, immune evasion, enhanced tumor angiogenesis, and strong abilities of invasion and metastasis [8]. The main reason for the poor prognosis of PC stems from the biological characteristics of early local invasion and distant metastasis of PC cells, which could be regulated by extracellular matrix (ECM) components [9]. One of the main clinicopathological features of PC is the existence of a large amount of fibrous tissue and abundant ECM around tumor cells [10]. Studies have shown that these special ECM components can not only provide physical support and protection to the tissue but also regulate the invasion and metastasis of PC by interacting with ECM receptors on PC cells [11,12,13,14]. Among those ECM receptors, integrins, the most extensively studied family, play an important role in controlling signal transduction during ECM–PC cell communications. This review will summarize the current knowledge about integrins, emphasizing integrin-mediated signaling pathways in PC, such as the outside-in and inside-out signaling. In particular, we will focus on the roles of the β1 subunit in the occurrence and development of PC and present strategies targeting integrin β1 for researching PC diagnostics and treatment.

## 2. Signaling Pathways Mediated by Integrins in PC

**Figure 1:**
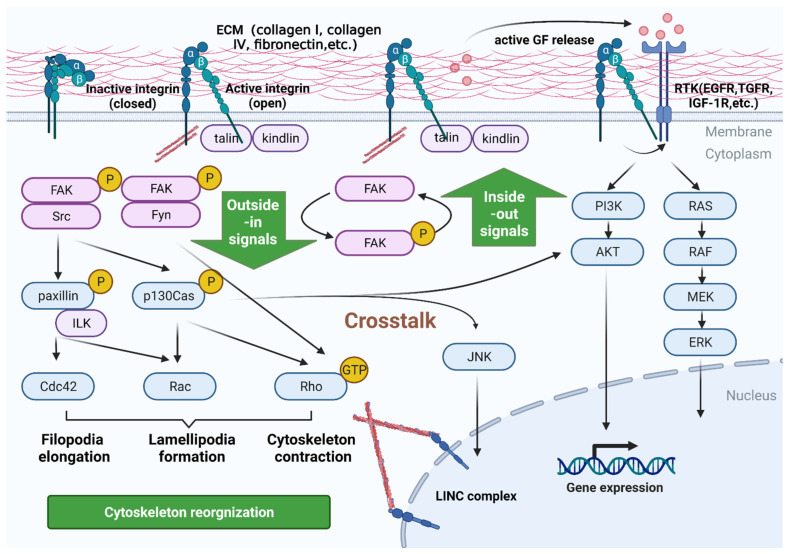
Signaling pathways mediated by integrins in PC cells. Integrins are heterodimeric cell-surface proteins consisting of α and β subunits. They have both inactive and active conformations that determine their binding affinities for ECM ligands (e.g., collagen I, collagen IV, fibronectin). Inactive integrins are always bent with physiological low-affinity, whereas active integrins are in an extended state with high-affinity and exhibit the separation of α and β lower legs. Active integrins recruit the adapter talins and, subsequently, activate FAK and Src. FAK/Src complex recruits and phosphorylates p130Cas and paxillin, and then induces the activation of downstream signaling, including Rac, Cdc42, and Rho, which are crucial regulators of cytoskeletal reorganization. This process can transmit information on their ligands’ chemical identity and physical state into cells, called the outside-in signals. Integrin adhesion can also crosstalk with other RTK pathways to regulate cell migration, survival, and growth. Additionally, integrins respond to inside-out signals by regulating protein interactions at the cytoplasmic tail region of integrins and modulating the strength of cell adhesion.

Integrins, a transmembrane glycoprotein molecule, are one of the members of the cell surface adhesion molecule family, and their primary biological role is to mediate cell-to-cell and cell-to-ECM interaction [15,16,17]. Regarding the structure, integrins respond specifically to ECM signals under different physiological/pathological conditions through their unique transmembrane bimolecular subunits α and β [18]. Up to now, 18 α and 8 β subunits have been reported in mammalian cells, forming 24 different integrins [19,20]. Among them, the subfamily composed of αvβ1, αvβ3, αvβ5, αvβ6, αvβ8, α5β1, α8β1, and αIIbβ3 is the most widely researched. Members of this subfamily can recognize the specific Arg-Gly-Asp (RGD) short peptide, which diffusely exists in the ECM rich in fibronectin, vitronectin, osteopontin, and fibrinogen [21]. Another subfamily consists of α1β1, α2β1, α3β1, α4β1, α6β1, α7β1, α10β1, α11β1, and α6β4, and exerts the effects by specifically recognizing collagen or laminin molecules in the ECM [22]. The conduction of integrin-mediated signal transduction needs the cooperation of α and β subunits, wherein the α subunit mainly recognizes specific ECM molecules. In contrast, the β subunit is responsible for transducing related cell signals. Abnormal expression of α or β subunits, or alterations in their synergistic effect, can lead to the occurrence and development of tumors [23,24]. Studies have shown that cancer cells obtain multiple biological behaviors in epithelial-derived solid tumors by expressing different integrins. In PC, various integrins have been reported to drive epithelial–mesenchymal transition (EMT), induce cancer stem cells (CSCs), facilitate metastasis, and promote treatment resistance. For example, PC cells are known to regulate CSC adhesion by expressing integrin α6 and β3; integrin αvβ3 can promote PC stemness and drug resistance and is a marker for PC [25]. As for tumor metastasis, integrin β3 was found to increase anchorage-independent cancer cell proliferation, thereby accelerating metastatic formations growth of PC [26,27]. Regarding radiochemoresistance, Jin et al. identified β8 integrin in three-dimensional (3D), ECM-based cell cultures as a potent therapeutic target for PC, and the mechanism involves integrin β8-induced autophagy upon irradiation [28]. Based on these fundamental research, targeted strategies have been explored for PC treatment. Moore et al. unveiled that peptide–drug conjugate SG3299, developed from foot-and-mouth disease virus, showed αvβ6-selectivity in vitro and in vivo and could specifically eliminate αvβ6-positive cancer cells, representing a promising approach for PC therapy [29]. Another phase I clinical trial utilized an αvβ3 targeting protein to selectively deplete cancer-associated pancreatic stellate cells, enhancing drug delivery and gemcitabine efficacy in PC by suppressing tumor angiogenesis [30]. The following section lists the main signaling pathways mediated by integrins in PC (Figure 1).

### 2.1. Bidirectional Signaling Transduction Controls the Coupling of Ligands with Cytoskeleton and the Formation of Focal Adhesion

As the main ECM receptor molecule on the cytomembrane, integrins activate key signaling intracellular molecules through the interaction with various ECM molecules, thereby regulating the expression of related genes and, ultimately, mediating biological behaviors such as cell proliferation, differentiation, movement, and migration, which are the so-called outside-in signals. The outside-in signaling refers to the fact that integrins bind to ligands in clusters and affect intermolecular or intramolecular connections inside the cell. With initial ligand binding, more and more adaptor proteins such as focal adhesion kinase (FAK) are recruited to the integrin cytoplasmic tail. They are phosphorylated, thereby progressively strengthening the focal adhesions. FAK associates with proto-oncogene tyrosine–protein kinase Src and becomes active. In the context of extracellular matrix adhesion, such plaques are called “cell-matrix adhesion” [31]. Integrin-mediated activation of the FAK/Src complex can control the activation of Rho family members, which are crucial regulators of cytoskeletal dynamics [32]. The activated FAK/Src complex first recruits and phosphorylates p130Cas, and then phosphorylated p130Cas recruits Dock180 and engulfment and cell motility (ELMO) through the adaptor protein Crk. The Dock180/ELMO complex can act as a guanine exchange factor (GEF) for Rac [33]. Rac GTPase is essential for Arp2/3-mediated growth of branched-chain F-actin, which drives membrane protrusions in the form of lamellipodia. Another cell-matrix adhesion protein, paxilin, can be phosphorylated by the FAK/Src complex. Paxilin recruits paxilin kinase linker (PKL) and Pak interacting exchange factor (PIX), two GEFs of Rac and Cdc42 (another Rho GTPase that drives filopodia elongation) [34,35]. However, when an external force is applied to integrins, FAK cooperates with another Src family kinase, Fyn, to activate two GEFs of RhoA, leukemia-associated RhoGEF (LARG) and GEF-H1, to enhance the contractility of the cytoskeleton, resulting in the reinforcement of the cell [36]. By coordinating the activity of Rho GTPases and the recruitment of components of the actin polymerization machinery, such as Arp2/3, the integrin-mediated adhesion complex is a signaling hotspot for the regulation of cytoskeletal dynamics. The inside-out signaling pathway is the intracellular signaling pathway that regulates protein interactions at the cytoplasmic tail region of integrins, which induce conformation changes in integrin extracellular domains. The inside-out signaling is regulated by talins and kindlins, [37], as well as the dephosphorylation and O-GlcNAcylation of the focal adhesion complexes [38]. In detail, talins and kindlins use the four-point-one, ezrin, radixin, and moesin (FERM) domain to connect with the tail of the integrin and trigger its conformational change for activation by separating the tail. If intracellular signaling disrupts intramolecular interactions within talins, the interaction between talins and integrins can be affected. The mode of regulation of kindlins is still unclear, and the coordinated behaviors of the two proteins are also illusive. Apart from talins and kindlins, the lateral diffusion and aggregation of integrins itself can further regulate cell adhesion strength [39]. In PC, many integrins have been reported to contribute to focal adhesion formation. For example, collagen I can facilitate adhesion, accelerate motility, and stimulate trans-migration through integrin α2β1. Mechanistically, collagen I induces the formation of F-actin and focal adhesions in PC cells, which is mediated by increased phosphorylation and subsequent activation of FAK signaling [40].

### 2.2. Integrins Are a Bidirectional Pressure Signal Transmitter

Integrins and some cell-matrix adhesion-related proteins could act as pressure sensors [41,42]. Extracellular stress is transmitted via integrins to the cytoplasmic proteins attached, thereby exposing binding sites for intracellular interactions. In response to pressure, a series of conformational changes occur in integrins, and related proteins present in cell-matrix adhesion are activated, including FAK, p130Cas, vinculin, etc. In turn, intracellular stress can also change the conformation of the integrin-related protein complex, allowing integrins to pull proteins such as fibronectin in the ECM. This may lead to solidification of the ECM, and once binding sites are exposed, more interactions between fibronectin molecules may occur during fibril formation or collagen network remodeling [43,44]. Thus, integrins allow cells to balance intracellular cytoskeleton contractility and extracellular matrix solidification. Zeltz et al. found that integrin α11 expression is upregulated in PC and demonstrated a moderate level of α11^+^ in myofibroblastic cancer-associated fibroblasts (myCAFs) associated with PC tumors. Using a function-blocking α11 antibody to inhibit cell adhesion to collagen I could hinder fibroblast-mediated collagen remodeling and delay the 3D migration rates of PC myCAFs. These results suggest that interaction between integrins and ECM is an essential factor during the process of collagen remodeling [45].

### 2.3. Crosstalk with Other Receptors in Inside-Out Signaling Pathways

The above-described integrin-regulated signaling pathways are not independent but act synergistically with signaling from other receptors. In fact, a major part of integrin signaling may involve the activation of other downstream receptor pathways. One of the earliest examples of this concept came from the study of the adhesion control of the Rac small GTPases. It has been shown that activating Rac in response to cell adhesion requires epidermal growth factor receptor (EGFR) signaling [46]. In this context, the ability of integrins to aggregate key enzymes, including kinases and GTPases and corresponding substrates, could trigger growth factor signaling through these enzymes. However, it is now evident that integrin-mediated adhesion can more directly lower the threshold for receptor tyrosine kinase (RTK) activation. Integrins can associate with several RTKs, including EGFR, insulin-like growth factor one receptor (IGF-1R), vascular endothelial growth factor receptor (VEGRF), platelet-derived growth factor receptor (PDGFR), hepatocyte growth factor receptor (HGFR/cMet), and macrophage stimulating one receptor (MST1R; Ron). In some studies, this integrin-RTK crosstalk has been proved to be mediated by Src family kinases [47,48].

Integrin-mediated adhesion to the ECM can enhance growth factor signaling in another way. Many growth factors are associated with heparin or heparan sulfate found in ECM proteoglycans [49]. Proteolytic cleavage of ECM proteins can release growth factors to bind to receptors, and, in some cases, interactions with the ECM have been found to contribute to the efficient presentation of growth factors to their receptors. ECM proteins also contain growth factor-like motifs such as TGFβ. In its inactive form, TGFβ is bound and masked by latency-associated peptide (LAP). Integrin αv can interact with the RGD motif in LAP and causes the exposure of activated TGFβ, which subsequently binds and activates the TGFβ receptor. Interestingly, this can occur through different protease-dependent or protease-independent mechanisms, the latter involving the integrin’s traction forces exerted on the TGFβ–LAP complex by the actin cytoskeleton [50,51]. Recent studies show that high expression of integrin αv in PC cells is associated with reduced survival in patients, and the knockdown of integrin αv in PC cells massively restrains primary tumor growth, peritoneal carcinomatosis, and pulmonary metastasis. The mechanism behind it demonstrates that integrin αv could activate latent TGFβ and thereby drives EMT [52]. Additionally, integrin αv is upregulated in macrophages and promotes the stemness of PC cells. Mechanistically, macrophage-expressed integrin αv facilitates the acquisition of stemness properties of PC cells by regulating the TGFβ/Smad2/3 pathway. These data indicate that strategies targeting the interaction between integrins and other receptors might be a new therapeutic modality for PC [53].

### 2.4. Control Signaling by Anchoring and Regulating the Cytoskeleton

By anchoring and regulating the cytoskeleton, integrins contribute to gene transcription while conceiving and responding to mechanical forces. This response can be achieved by altering the concentrations of second messengers such as calcium and cyclic adenosine and crosstalk with the growth factor receptor signaling pathway, as mentioned above [54]. More directly, the cytoskeleton is linked to integrins on the plasma membrane as well as the nuclear membrane through linkers of the nucleoskeleton and cytoskeleton (LINC) complex. Here, the nesprin proteins in the outer membrane connect to microtubules, actin fibers, and intermediate filaments, while the SUN protein in the inner membrane binds to the nuclear lamina. Since chromatin-binding proteins and DNA are attached to the nuclear layer, extracellular mechanical stress may be propagated to chromatin and cause gene expression through conformational modifications of DNA and related proteins [55]. However, currently, direct evidence demonstrating a purely mechanical coupling between the ECM and gene expression is still lacking. In a previous study, integrin α2 was shown to be overexpressed in PC and promoted the proliferation and invasion abilities of cancer cells. RNA-seq assays indicated that integrin α2 transcriptionally regulated the expression of PD-L1 in PC, which was further shown to be mediated by the STAT3 pathway [56]. Thus, interfering with integrin-mediated gene transcription such as PD-L1 is a feasible method to enhance the efficacy of checkpoint immunotherapy against PC.

## 3. An Overview of Integrin β1

Integrin β1, also known as CD29, is a human protein-coding gene with a full length of 58048bp. Located on human chromosome 10p11.2 with a total of 18 exons, it has three transcript variants named transcript variants 1A, 1E, and 1D. Transcript variant 1A has a full length of 3735bp with 16 exons, and encodes a protein of 798 amino acids; transcript variant1E has a full length of 3794bp and encodes a protein of 798 amino acids; transcript variant1D has a full length of 3739bp and encodes a protein of 80l amino acids [57].

As the most common β subunit of the integrin family, integrin β1 is currently known to bind to different α subunits to form 11 different integrins. Among these, integrin β1 forms integrins very late antigen (VLA)-1 to 6, α7β1, α8β1, and αvβ1 (vitronectin receptor, VNR) with integrins α1 to α8 and αv subunits, respectively, constituting the VLA family of integrins, which is widely distributed throughout the body [58]. A series of studies have unveiled that integrins composed of β1 subunit play a key role in maintaining the stemness property of tumor cells as well as promoting tumor metastasis and chemotherapy/radiation resistance by participating in the transduction of various intracellular signaling pathways [59,60,61,62,63,64,65]. In PC, the β1 subunit has been closely related to the vascular invasion, distant metastasis, and survival of the patients, and treatment targeting integrin β1 in PC has gained initial success in animal models [66]. Table 1 summarizes the studies regarding integrin β1 in PC.

## 4. Roles of Integrin β1 in the Malignant Behaviors of PC

### 4.1. Integrin β1 and Proliferation-Related Signaling

Integrins are critical for cell proliferation under physiological and pathological conditions [67]. As for the behind mechanism, interactions between integrins and growth factor receptors (GFR) have drawn increasing attention these years. In normal cells, diverse receptors including EGFR, cMet, PDGFR, and VEGFR can interact with integrins to activate cell proliferation activity. Previous studies found that integrins could directly activate EGFR in normal endothelial cells independent of EGF. Experiments in liver regeneration demonstrated that the downregulation of the β1 subunit in hepatocytes decreased c-Met and EGFR phosphorylation, thereby inhibiting cell proliferation [68,69]. In tumor cells, integrins also play a part in cell proliferation by interacting with GFR [70]. In vivo/in vitro experimental studies discovered the interaction between EGFR and integrin α5 as well as integrin β1 in tumors [71,72]. Studies in hepatoma have found that the cellular macromolecular protein cysteine-rich protein 61 (CYR61/CCN1) can stimulate the accumulation of reactive oxygen species (ROS) in cells by interacting with integrin α6β1, thereby inhibiting the activation of the EGFR signaling pathway and the proliferation of hepatoma cells; in addition, using siRNA to interfere with the expression of the integrin β1 subunit could effectively suppress the viability of hepatoma cells [73,74]. Regarding PC, overexpression of integrin β1 and the downstream Src-AKT activation have been reported. This triggers an EGFR ligand-independent proliferation signaling, bypassing the EGFR-blocking effect, and mediates resistance to the anti-EGFR monoclonal antibody, cetuximab. Knockdown of integrin β1 or inhibition of Src or AKT can successfully re-sensitize cetuximab-resistant (CtxR) PC cells to cetuximab. The researchers then discovered that neuropilin-1 (NRP1) physically interacted with active integrin β1, but not the inactive one on the cell surface. They generated an EGFR and NRP1 dual targeting antibody, Ctx-TPP11, to simultaneously inhibit active integrin β1-driven signaling and suppress EGFR signaling. Further experiments proved the efficacy of Ctx-TPP11 on the inhibition of PC proliferation, both in vitro and in vivo. This study offered an effective therapeutic strategy based on EGFR and integrin β1 dual targeting, which might become a hot topic in PC therapy [75].

### 4.2. Integrin β1 and Tumor Suppressor p53

Numerous studies have reported the inactivation of the tumor suppressor protein p53 in different forms of human cancer [76]. It is believed that the leading cause for the inactivation of the wild-type p53 signaling pathway in tumor cells is the deletion mutation of the p53 gene or abnormal upregulation of its inhibitory proteins, among which integrins play an indispensable role [77,78].

Previous literature demonstrates that tumor cells can suppress p53 activation through integrin α5β1 in response to chemotherapeutic drugs, thereby downregulating the drug sensitivity [79]. In breast cancer, inhibiting integrin α2β1 can upregulate the expression of wild-type p53. At the same time, glioma cells can inhibit the expression of wild-type p53 by upregulating integrin α5 to enhance tumor chemotherapy resistance [80]. Recent studies have found that the integrin α5β1/AKT/PEA15/caspase8 signaling pathway in glioma can directly regulate the activity of p53. Integrins can also interact with p53 through the downstream protein kinase focal adhesion kinase (FAK), thus inhibiting its activity [81]. In PC, integrin β1 is also found to mediate mutant p53-driven cancer invasion, which needs the facilitation of a filopodia-inducing motor protein, Myosin-X (Myo10). Experiments using a Myo10 mutant without the integrin-binding domain revealed that the ability of Myo10 to transport integrin β1 to the filopodia tip is required for invasion. The introduction of mutant p53 promoted Myo10 expression in a mouse PC model, whereas suppression of endogenous mutant p53 attenuated Myo10 levels and cell invasion. These results suggest that cell components that contribute to plasma-membrane protrusions, such as integrin β1, may serve as specialized metastatic engines for mutant p53-driven PC [82]. It is believed that activation of p53 function is a vital strategy to intervene in tumor progression [83]. The studies mentioned above imply that inhibiting the activity of integrins and their downstream signaling pathways will provide a new approach to activating p53 and will become a novel research orientation in the field of tumor therapy.

### 4.3. Integrin β1 and Cell Apoptosis

Regulation of the normal cell cycle via interaction of integrins with ECM is crucial in promoting embryonic development and maintaining tissue homeostasis. The imbalance of this interaction and the resulting inactivation of the downstream PI3K/AKT, MEK/ERK, FAK, NF-κB, and ILK signaling pathways could trigger abnormal cell apoptosis [84]. During tumor metastasis, due to disengagement from the interaction with ECM, cancer cells exhibit an enhanced ability to resist apoptosis by reprogramming the expression of integrins [85]. In hepatoma cells, upregulation of miR-26a can inhibit the expression of integrin α5 and promote tumor apoptosis [86]. It has been reported that melanoma cells express the matrix metalloproteinase inhibitor 1 (TIMP1) to resist apoptosis by forming complexes with CD63 and integrin β1 [87]. In breast cancer, upregulation of integrin α6β1 can decrease the expression of non-receptor tyrosine kinase FER in the cytoplasm, thereby impairing the ability to resist apoptosis [88]. In addition, vacuolar–ATPase inhibitors have been shown to regulate the anti-apoptotic ability of various tumor cells by reducing the activity of integrin β1 [51], and the zinc finger transcription factor ZNF304 can enhance the resistance to cell apoptosis by regulating integrin β1 transcription [89,90]. In PC, integrin β1 is also reported to be involved in apoptosis. Notably, numerous materials have been identified to regulate this process. For example, methylseleninic acid can induce entosis by cell detachment through downregulation of Cdc42 and integrin β1, and fucoxanthinol (FxOH) suppresses apoptosis of PANC-1 cells by upregulating the expression of integrin β1, FAK, paxillin, FYN, AKT, and PPARγ [91,92].

### 4.4. Integrin β1 and Angiogenesis

The involvement of integrins in regulating angiogenesis under various conditions has been extensively investigated. In tumor therapy, αvβ3/β5 is the first group of integrins identified to have the function of promoting the growth of new tumor vessels, and its functional antagonist cilengitide is also the first anti-tumor angiogenesis drug used in clinical research [93]. Unfortunately, cilengitide failed to improve overall survival in a multicenter, randomized, controlled phase 3 study in glioma [94]. Subsequent studies suggest that the antitumor effect of cilengitide is closely related to the time and dose of administration, and different conditions may lead to opposite results [95]. α5β1 is another integrin that promotes tumor angiogenesis [70,96]. Studies on the molecular mechanism behind the β1 subunit regulating angiogenesis found that angiopoietin-2, Arf6, VE-cadherin, and MAP4K4 were involved in activating the β1 signaling pathway, thereby regulating the subsequent angiogenesis process [97,98,99,100]. Integrins can accelerate the growth of new blood vessels and enhance the resistance to anti-angiogenic drugs. Bevacizumab, a commonly used anti-tumor angiogenesis drug, has shown promising effects in treating various tumors [101]. In clinical practice, however, drug resistance emerged during the treatment of glioma with Bevacizumab. Studies have detected that the expression of integrin α5β1 and its ligand fibronectin in drug-resistant tumor cells is significantly increased, while interfering with the expression of β1 can restore the sensitivity of tumor cells to Bevacizumab and improve the therapeutic efficacy [102,103]. While anti-angiogenic drugs have not been applied in the treatment of PC, research on the engagement of integrin β1 in PC angiogenesis has also achieved progress. Studies reveal that the loss of integrin β1 binding to fibulin-5 (Fbln5) can inhibit tumor angiogenesis in endothelial cells by increasing the level of ROS, suggesting that blocking Fbln5 function or interaction between Fbln5 and integrin β1 could be an effective anti-tumor strategy, alone or in combination with other therapies [104].

### 4.5. Integrin β1 and Metastasis

Early metastasis is a hallmark of PC pathology. The entire metastatic process involves the following distinct steps: EMT, invasion, intravasation (from primary tumor sites to enter blood vessels or lymphatic vessels), extravasation (from circulation to distant metastasis sites), and colonization to form secondary malignant tumor [105,106]. During this process, the roles of integrin β1 go all from the beginning to the end [107,108]. By activating integrin β1, the HGF/c-Met signaling pathway can promote the EMT transformation of gastric cancer [109,110,111,112]. Sheng et al. found that EGF simultaneously induced EMT and activated the integrin β1/EGFR-ERK/MAPK signaling pathway in three PC cell lines. This pathway could be further regulated by Calreticulin (CRT). Immunofluorescence showed that CRT was co-stained with pEGFR1173, fibronectin, and integrin β1 in PC cells, and overexpressing CRT reverted the change in EMT-related proteins induced by EGF. These results indicate a crucial function of the integrin β1/EGFR-ERK/MAPK axis signaling pathway in the EMT of PC [113].

Early studies show that tumor cells degrade and remodel the ECM by regulating the expression of matrix metalloproteinases (MMPs) through integrin signaling, thereby promoting invasion [114,115]. In PC, such effects of integrin β1 are executed by MMP-2. Eukaryotic elongation factor-2 kinase (eEF-2K) is an atypical kinase that is highly upregulated in PC cells. Researchers found that downregulation of eEF-2K impaired the invasion of PC cells and significantly decreased the expression of tissue transglutaminase (TG2), a multifunctional enzyme implicated in the regulation of cell attachment, motility, and survival. These alterations were associated with reductions in β1 integrin/uPAR/MMP-2 expressions and suppression in Src activity. Meanwhile, the induction of EMT biomarkers was also compromised by this axis, as demonstrated by the alterations of the zinc finger transcription factors, ZEB1/Snail, and the tight junction protein claudins. Therefore, the β1 integrin/Src/uPAR/MMP-2 signaling pathway represents a novel potential therapeutic target for PC invasion and EMT [116].

Apart from facilitating invasion, matrix proteolysis is also engaged in tumor cell intravasation. The behind mechanism involves the production of growth factors and cytokines, which stimulate neo-angiogenesis [117]. After circulation in the blood, the next critical step for tumor cells is extravasation. Integrins expressed on both cancer cells and endothelial cells have implications in extravasation. It has been illustrated that endothelial integrin α5 can bind to neuropilin 2 (NRP2), a multi-functional non-kinase receptor for diverse growth factors expressed on cancer cells, mediating extravasation. In the mouse PC model, by interacting with integrin α5 on the endothelial cell, the PC cell can bind to the endothelium and accomplish vascular extravasation [118]. Regarding colonization after extravasation, research shows that blockage of activated integrin α5β1 inhibits both lung and bone colonization of breast cancer cells [119]. Although similar experiments in PC have not been reported, these studies demonstrate that integrins, especially integrin β1, are in close relationship with tumor metastasis, and, therefore might become a critical target for suppressing PC progression.

### 4.6. Integrin β1 and Tumor Microenvironment (TME)

As cancer develops, it causes alterations in the surrounding tissue to create a favorable tumor microenvironment (TME) for its successful growth. It mainly includes ECM, surrounding blood vessels, immune cells, fibroblasts, and various signaling molecules [120]. It is currently believed that integrins specifically expressed on the cell surface and the corresponding composition of ECM in the tumor microenvironment are the key factors that determine the distant metastasis of tumor cells [121]. For example, liver metastasis of colon cancer depends on whether tumor cells express integrins α2β1 and α5β1 that facilitate cell survival in the liver microenvironment [122]. Similarly, cancer cells metastasize to the bone via the expression of integrins αvβ3, α2β1, and α4β1 that bind to specific ligands in the bone ECM [123]. Pancreatic stellate cells (PSCs) are the most abundant stromal cell types in PC. They are a major source of tumor-associated fibroblasts (CAFs) that can be activated through growth factors secreted by cancer cells. Collagen type V, expressed by PSCs, can affect the malignant phenotype of various PC cell lines, and stable downregulation of collagen type V in PSCs could reduce metastasis in a PC mouse model. This was further shown to be mediated by β1 integrin signaling, since pharmacological and antibody-mediated inhibition of β1 integrin signaling abolished collagen type V-induced effects on PC cells [124]. Integrins β1 are also expressed in CAFs. Studies on non-small cell lung cancer demonstrate that knocking out integrin α11β1 in CAFs can interrupt the interaction between tumor cells and CAF and ultimately block the distant metastasis of lung cancer [125]. In PC, galectin 3 (GAL3), a β-galactoside-specific lectin, contributes to PC development by stimulating IL8 transcription through integrin β1 on PSCs, further activating NF-κB through integrin-linked kinase (ILK). Thus, inhibiting integrin β1 expression on PSCs can potentially block PC growth [126]. Regarding immune response, it has been illustrated that upregulation of integrin αv expression can lower the sensitivity of tumor cells to immune attack caused by chemotherapeutic drugs [127,128]. Additionally, through the synergistic effect of integrin α5β1 and the extracellular matrix tenascin C, tumor cells can avoid the infiltration and attack of the surrounding immune cells [128]. In vivo experiments found that upregulation of the expression of integrin α4β1 can stimulate the activation and infiltration of T lymphocytes in the tumor tissues, thereby restraining tumor growth [129]. Recent research has found that normal immune cells can promote tumor metastasis in a specific environment, and it is speculated that the mechanism could be that tumor cells might enhance the invasion and metastasis activities by interacting with integrin αMβ2 contained in exosomes secreted by immune cells [130]. Research on the regulation of PC tumor immunity by integrin β1 is still lacking, but this may hopefully become a future direction for further investigation.

### 4.7. Integrin β1 and CSCs

Recently, a subpopulation of cells with self-renewal and differentiation abilities, termed CSCs, has been described and is assumed to be the driver for malignant characteristics by engaging in the processes of tumor growth, metastasis, and drug resistance [131,132,133]. CSCs are often identified with an expression of stemness markers including CD24, CD44, Nanog, CD133, Sox2, Sox9, essential specific antigen (ESA), and Kruppel-like factor 4 (KLF4) [134,135,136]. Integrins have been illustrated to play a pivotal part in cancer initiation, progression, and differentiation, indicating their contribution to CSC properties in diverse human cancers, including PC [137]. Barnawi and his colleagues analyzed the expression profiles of β1 integrin in 530 breast cancer patients and reported a correlation between β1 integrin and fascin expression; further research demonstrated that fascin facilitated the abilities of adhesion, self-renewal, and chemoresistance in breast cancer cells through β1 integrin [138,139]. In mice lacking β1-integrin function, complete inhibition of tumorigenesis was observed; in the mammary gland, tissue-specific loss of function of β1 integrin can effectively abrogate the generation and proliferation of CD24^hi^CD29^lo^CD61^hi^ cancer cells [140,141]. Studies in squamous cell carcinoma reveal that α6^hi^β1^hi^ cells can initiate secondary tumors while those with α6^lo^β1^lo^ expression cannot, providing evidence for integrin β1-mediated CSC properties [142]. In PC, researchers isolated CD24^+^CD44^+^ stem-like cells from the PANC-1 cell line and proved increased invasion ability of these cells compared to CD24^-^CD44^+^ cells. Using lectin microarray and nano LC-MS/MS, they identified upregulated integrin β1 expression in CD24^+^CD44^+^ stem-like cells [143]. Mechanistically, PC cells can activate CAFs and increase collagen synthesis, which further leads to enhanced PC self-renewal and migration, as well as increased frequency of CSCs through FAK activation. Inhibition of the integrin β1/FAK signaling in PC cells significantly blocked the impact of CAFs on clonogenic growth [144]. Another research work reports that pancreatic CSCs express elevated aldehyde dehydrogenase (ALDH), which are associated with metastatic property [145]. β1 integrin–FAK expression was enriched in these ALDH+ CSCs, and further FAK inhibition abrogates clonogenic PC growth in vitro and in vivo [146]. These findings demonstrate that β1 integrin enhances CSC properties and promotes tumor initiation, self-renewal, and metastasis through FAK signaling. Therefore, targeting integrin β1 may potentially be applied as a potent approach for PC treatment to restrain CSC survival and aggressiveness.

### 4.8. Integrin β1 and Therapy

Surgical resection remains the preferential approach for PC in the early stages. In contrast, for advanced PC, radiotherapy and chemotherapeutic agents, including gemcitabine (Gem), nab-paclitaxel, 5-fluorouacil (5-FU), and FOLFIRINOX, are generally recommended as adjuvant options. Despite the great progress made in these strategies, the overall survival (OS) of PC patients is still unsatisfactory due to the generation of chemoresistance or radioresistance [147]. Apart from actions on tumor pathogenesis and progression, increasing numbers of data show that integrins also play important roles in resistance to treatment. Patients with higher levels of integrin β1 tend to be more resistant to chemotherapy and display a worse clinical outcome [148]. We will discuss various factors that are more or less involved in integrin β1-mediated therapeutic resistance.

The extensive desmoplastic reaction is a prominent pathological feature of PC and shapes a physical barrier for drug delivery. Under the control of growth factors secreted by PC cells, PSCs can be activated and are responsible for dense ECM deposition, which, in turn, regulates resistance to standard therapies through interaction with tumor cells based on various adhesion molecules, with integrins being the largest family [149,150,151]. A previous study demonstrated that under treatment with 5-FU and Gem, PC cells cultured on collagen V-coated plates exhibit significantly increased survival rates compared to the controls, which can be reversed by inhibiting the integrin β1 signaling pathway [124]. In 95% of PC cases, activating mutations in the KRAS oncogene are detected, but clinical agents that directly target mutant KRAS are, so far, not available. Nevertheless, inhibition of downstream effectors, including the MAPK signaling pathway and PI3K signaling pathway, has received increasing attention these days [152,153]. In a 3D culture model of PC, MEK inhibition induced apoptotic lumen formation, a single-layered cluster with the cells at the periphery of the cluster displaying resistance to MEK inhibition while the cells in the interior layers undergo apoptosis. Following administration of the integrin β1 neutralizing antibody, the cells in the matrigel matrix were scattering, and survival in the context of MEK inhibition significantly decreased [154]. Taken together, these data suggest the pivotal role of integrin β1 signaling in the treatment resistance of PC induced by interaction with ECM.

Cumulative evidence supports that integrins can also contribute to drug resistance by interacting membrane proteins other than ECM. For instance, integrin β1 and Caveolin-1(Cav-1), a cell membrane component protein at Caveolae, co-participate in cell motility, invasion, and chemoresistance in lung cancer as well as PC [155,156]. Notably, the radiosensitivity of PC cells was enhanced, and integrin β1 expression was significantly reduced after Cav-1 silencing [157,158]. Additionally, integrins can also crosstalk with GFR and transactivate RTK signaling, even in the absence of growth factor ligand, which indicates that integrin signaling has a relationship with acquired resistance to molecularly targeted agents such as Cetuximab, which was discussed earlier in this manuscript [150] [159]. Regarding the crosstalk mechanism between EGFR and integrins, FAK plays a prominent role in many similar signaling pathways that integrins share with EGFR. At the molecular level, FAK is phosphorylated and activated at distinct domains interacting with various cytoplasmic proteins such as paxillin, Src, PI3K, Grb2, and many others, initiating several downstream signaling pathways [160,161,162]. It is worth noting that through the assembly of FAK-Src-p130Cas complex, the JNK signaling pathway can be activated, leading to negative regulation of equilibrative nucleoside transporter 1 (ENT1) [162,163]. The ENT1 is well known for mediating gemcitabine intracellular transport and resistance in humans and has, therefore, been proposed as an attractive potential prognostic biomarker for gemcitabine response in PC [164,165,166]. A recent study showed that increased α3 and β1 expression and subsequent activation of integrin α3β1 signaling via JNK inhibit the expression of ENT1, which reduced gemcitabine uptake and accumulation into PC cells [167].

**Figure 2:**
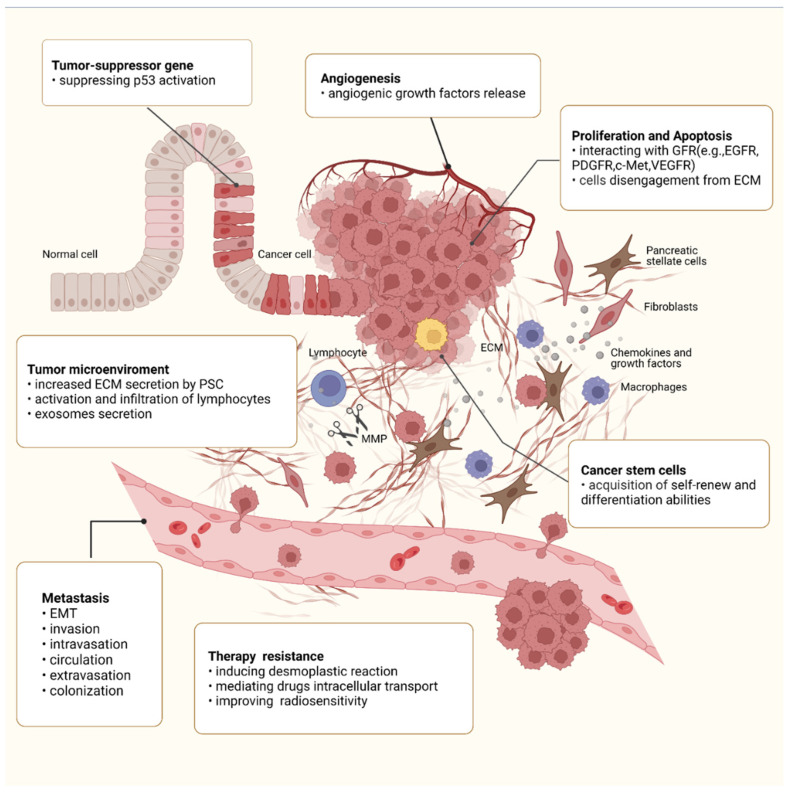
Roles of integrin β1 in the malignant behaviors of PC. Integrin β1 is involved in every step of PC progression and is responsible for regulating malignant cell behaviors such as sustained proliferation, apoptosis resistance, angiogenesis, and migration, as well as CSC property. Integrin β1 also promotes PC metastasis, including EMT, invasion, intravasation, circulation, extravasation, and colonization. In the tumor microenvironment, integrin β1 has been implicated in extensive desmoplastic reaction and regulates the expression of MMPs, the release of GF, and the activation and infiltration of lymphocytes. In addition, integrin β1 also plays an important role in the acquisition of treatment resistance.

Apart from drugs, integrin β1 has also been reported to be involved in cell-adhesion dependent radioresistance via direct contact between PSCs and PC cells. Mantoni et al. demonstrated, for the first time, that PSCs promote radioprotection of PC cells under a direct coculture condition rather than a conditioned medium from PSCs, which is attributed to the integrin β1-FAK signaling activation [168]. A further study, conducted by Mohamed et al., also confirmed the role of FAK signaling in improving PSCs-dependent radiosensitivity of cancer cells. The results showed that the FAK–tyrosine kinase inhibitor, VS-4718, can sensitize PC cells for radiation only in the presence of ECM-producing PSCs. The combination of VS-4718 and radiotherapy significantly reduced the growth of tumor aggregates in the 3D multicellular tumor model [169]. These effects may be attributed to impaired DNA repair, arrested cell cycle, and enhanced PC stem cell function [144,169,170]. A schematic diagram of the roles of integrin β1 in the malignant behaviors of PC is shown in Figure 2.

## 5. Clinical Significance of Integrin β1 in PC

Thus far, since no other effective screening techniques or biomarkers other than CA19-9 have been identified for PC, most patients (80–85%) are already in an advanced stage when diagnosed, and the survival rate remains low [171]. Therefore, the need to explore more reliable biomarkers with adequate sensitivity and specificity for the diagnosis and prognosis of PC is urgent. Numerous studies have demonstrated the abnormal expression of integrin B1, which is closely related to the progression, metastasis, and prognosis of PC. Here, we present a list of recent studies researching the feasibility of integrin β1 as a diagnostic and prognostic biomarker in PC (Table 2).

Recently, a bioinformatic analysis showed that the expression of integrin β1 is remarkably increased in PC tissue samples compared with normal pancreas specimens based on TCGA and Oncomine databases. The ROC curve revealed that integrin β1 functions as a crucial indicator for PC diagnosis, with an AUC value of 0.86. Furthermore, the assessment of integrin β1 expression in different stages of PC demonstrated that patients in the late stage present higher expression of integrin β1 than those in the early stage [172]. However, upregulation of integrins is also reported in non-tumor benign diseases of the pancreas. A study by Chen et al. illustrated that integrin β1 was also overexpressed in chronic pancreatitis tissue, but to a lower degree compared with PC [173]. These findings suggest that integrin β1 might not be suitable as an early diagnostic indicator, but is of particular significance for assessing the progression and prognosis of PC.

Despite the relatively high clinical value of tissue-expressed integrin β1, the specimens mentioned above were obtained surgically, making it inconvenient and unacceptable for early screening and detection of PC. Therefore, methods through specimens, which can be easily accessed, are much more worth expecting. In 30 preoperative PC patients, the expression of integrin β1 mRNA in peripheral blood mononuclear cells (PBMCs) was significantly higher than that in healthy individuals. Moreover, the mRNA level of integrin β1 was significantly associated with the clinical stage and liver metastasis of PC [174]. The significant correlation between integrin β1 protein expression in PBMC and clinical features in PC patients was further confirmed by subsequent analysis [175].

In addition to diagnosis, there is ample evidence assessing integrin β1 regarding the prognosis of PC patients. In a previous study, integrin β1 expression in 68 cases of PC tissues was investigated by immunohistochemistry (IHC), verifying the positive integrin β1 staining in the cytoplasm and cytomembrane of PC cells and describing a positive association between integrin β1 overexpression and UICC stage of PC patients. Importantly, Kaplan–Meier survival analysis proved that overexpression of integrin β1 contributed to the poor prognosis of these PC patients [113]. Another study included 54 PC patients who received no other treatment, such as chemotherapy, radiation therapy, or immunotherapy, before and after cryosurgery and analyzed the dynamic change of integrin β1 expression in PBMC after cryosurgery. They found that integrin β1 expression decreased dramatically in the later stage, especially three months after treatment. However, the mRNA and protein levels of integrin β1 increased ten days after cryosurgery, which may be attributed to the destruction of tumor cells in the initial stage after cryosurgery. Moreover, the results indicate that the expression of integrin β1 mRNA and protein was correlated with disease-free survival (DFS) and overall survival (OS) in PC, and they could function as independent prognostic factors for OS but not for DFS. These data suggest that integrin β1 expression in the blood may become a promising biomarker for evaluating the outcome of cryosurgery in PC patients [175,176]. Additionally, a retrospective analysis of 63 primary PC patients who received adjuvant gemcitabine chemotherapy after pancreatectomy shows that OS and DFS were shorter in patients with high integrin β1 staining than in those with low integrin β1 staining [148]. Similar results were observed in another recent study. In this study, the researchers examined the prognostic significance in 102 PC patients who received adjuvant chemotherapy or chemoradiation therapy after resection. They found that patients with overexpression of integrin β1 had significantly shorter postoperative survival time than those with low integrin β1 levels. Notably, the combination of integrin β1 with PODXL/BCL7B more accurately predicted the postoperative outcomes of PC patients, which were superior to the UICC TNM staging system [177]. In conclusion, these findings indicate that integrin β1 is an independent predictor of worse postoperative survival of PC and could be used as a reliable biomarker for forecasting the response to adjuvant therapies prior to their initiation.

## 6. Future Expectations

Apart from apoptosis, autophagy, necroptosis, and pyroptosis, ferroptosis is a new type of programmed cell death that is iron-dependent. The primary mechanism of ferroptosis is that, under the action of ferrous iron or ester oxygenase, unsaturated fatty acids highly expressed on the cell membrane are catalyzed for lipid peroxidation, thereby inducing cell death. In addition, decreased expression of the antioxidant system, including glutathione and glutathione peroxidase 4 (GPX4), is another manifestation of ferroptosis [178,179]. Previous studies report that the α6β4 integrin saves adherent epithelial and carcinoma cells from ferroptosis induced by erastin and ECM detachment [180]. The mechanism behind this involves suppressing proferroptotic membrane lipids through ACSL4, which is an enzyme that promotes ferroptosis by enriching membranes with long polyunsaturated fatty acids. Further experiments unveiled that this suppression is achieved by α6β4-mediated activation of Src and STAT3 [181]. This link between α6β4 and ferroptosis implicates potential strategies for cancer therapy. Thus far, treatment targeting α6β4-mediated ferroptosis has not been reported, but integrins could serve as a guide for ferroptosis-based drugs. For example, Xu et al. report the facile construction of a multifunctional theranostic nanoplatform based on doxorubicin (DOX)-loaded tannic acid (TA)-iron (Fe) networks (for short, TAF) coated with fibronectin for combination tumor chemo-/chemodynamic/immune therapy. With the help of fibronectin, this platform can specifically target tumor cells with high expression of αvβ3 integrin [182]. Similar drugs have also been developed for ferroptosis therapy of orthotopic brain tumors [183]. Research on integrin β1-associated ferroptosis in PC is not available to date. Still, considering the function of integrin β1 in cell attachment, we infer that integrin β1 might play a role in ferroptosis, and related therapies could be further expected.

Apart from existing on cell membranes of cancer cells and stromal cells, integrins are also contained in exosomes. Exosomes are late endosome-derived small extracellular vesicles (EVs) that assist intercellular communication under various conditions [184]. As a carrier for multiple molecules, including nucleic acid fragments, proteins, and lipids, exosomes play a vital role in cancer progression [185,186]. Several studies have verified the role of exosomal integrins in PC. For example, integrin β4 increases PC growth, migration, and invasion by regulating plectin inclusion in the exosomes; exosomal integrin αvβ5 supports liver metastasis by interacting with fibronectin-rich ECM [187,188]. Another study found that loss of protein kinase D1 contributed to metastasis of pancreatic tumors to the lung in mice by increasing the release of EVs, which showed upregulated loading of integrin α6β4. However, the specific role of α6β4 here still awaits further exploration [189]. Although Casari et al. demonstrate that integrin β1 was equally expressed in malignant vs. non-malignant exosomes, it does not mean exosomal integrin β1 is not involved in PC progression, and more profound research is needed to clarify the possible association [190].

Thus far, numerous preclinical models have indicated that integrin β1 is of potential therapeutic benefit in PC alone or in combination with the standard of care. Nevertheless, clinical studies have not received encouraging results. Even for the whole integrin family, most integrin-targeting strategies failed to reach the market, mainly due to a lack of therapeutic efficacy and safety. Volociximab is a chimeric monoclonal antibody against integrin α5β1 and can prevent neovascularization and suppress tumor growth and metastasis in preclinical studies [191]. Unfortunately, a multicenter, two-cohort phase II trial illustrated that a combination treatment of gemcitabine with volociximab did not enhance treatment efficacy over monotreatment in patients with metastatic PC. Moreover, volociximab has thus far failed to show benefit for patients with melanoma, ovarian cancer, or non-small cell lung cancer [192,193]. In addition to volociximab, several other monoclonal antibodies against integrin β1, such as OS2966 and P5, have been reported in clinical trials. Specifically, OS2966, a first-in-class, humanized, and de-immunized monoclonal antibody anti-β1 integrin which is tested in patients with high-grade glioma (HGG) [194], and P5, a pan-β1 murine monoclonal antibody which is claimed to act at α5β1 predominantly, are reported in PH3 trials for NSCLC [195]. However, no clinical trials regarding these anti-pan-β1 antibodies have been tested in PC, and whether they deserve further testing in PC is an open question. In regards to other integrin β1 inhibitors, such as ATN-161 and natalizumab, no clinical trials have been carried out in PC, though they have already failed to yield satisfying outcomes in other solid tumors [196]. Obviously, it is challenging to translate the preclinical data on integrin β1 into clinically efficacious drugs considering the complex and wide-ranging roles of integrin β1 in tumors.

Several factors have complicated the development of integrin-based therapeutics for cancer [197,198]. First, many integrins exhibit an overlapping ligand-binding spectrum with other integrin heterodimers. Another integrin binding the same ligand can compensate for the effect of blocking one integrin with a single agent. This functional redundancy and compensation between different integrins could explain the good tolerance but limited therapeutic effects of these integrin inhibitors [191,192]. Second, the knowledge of integrins’ different roles in distinct disease models and stages is far from being understood. Patients enrolled in the clinical trials are always in advanced disease stages and often have extensive treatment histories. The integrin expression patterns may have changed and become complicated with a mix of primary and metastatic lesions. For example, while depletion of integrin β1 suppresses the progression of several tumors, it could enhance tumorigenesis and proliferation in prostate cancer [196]. Likewise, Moritz et al. present a dual role of integrin α2β1 in breast cancer, that inhibiting α2β1 expression may be beneficial to limit the expansion of primary tumors but could be harmful once tumors establish in bone [199]. Besides, it has been reported that some antagonists can, to some extent, be agonists under certain conditions and induce the receptor to extend and adapt to a high-affinity ligand-binding state, which may paradoxically enhance angiogenesis and tumor growth [200,201,202]. Third, most integrin-based therapeutics in clinical trials, including antibodies or peptides, typically block integrin function by occupying the ligand-binding site. During conformational changes, the bent form corresponding to a low-affinity conformer can also engage ligands without inducing activation signals [203]. Moreover, compared to allosteric inhibitors, which can lead to rapid dissolution of the integrin–ligand complex, integrin antagonists are unable to disrupt pre-existing integrin–ligand interactions, which may be one of the possible explanations for the lack of efficacy in the clinical trials [204]. Thus, allosteric inhibitors could also be powerful adjuvants to existing integrin therapies. Fourth, the lack of suitable preclinical models which can accurately represent human biology is a general problem in drug testing. It is challenging to correlate preclinical models with the actual clinical situation of patients with advanced and metastatic disease. Fifth, safety and tolerability are critical factors that cannot be ignored in the translation of preclinical data. Given the crucial role of integrin β1 in maintaining the integrity of the skin and upper gastrointestinal tracts, targeting the entire β family may not be achievable due to the interference with normal physiological processes and unacceptable toxicity [150]. Furthermore, it is worth noting that integrin β1 is a significant carrier of *N*-glycans, which are thought to play crucial roles in many biological functions [205]. *N*-glycans are complex forms of post-translational modifications (PTMs) and exhibit different structures in a space and time-dependent manner. Thus, the structural heterogeneity of *N*-glycans on integrin β1 among different individual patients may also be a non-negligible factor that affects the inhibition efficiency of β1, which leads to the failure of integrin β1-targeted therapeutics in clinical trials. In short, all the above mechanisms may lead to the failure of integrating integrin-targeting treatment in PC. Therefore, a deeper exploration of the efficacy and feasibility of targeted drugs needs to be conducted to overcome these obstacles in the future.

Since integrin-targeted inhibitors experienced setbacks in clinical trials, peptides targeting integrins are being proposed for their usefulness in drug delivery and non-invasive tumor imaging [206,207]. A functionalized liposome decorated with the integrin α5β1 binding peptide PR_b has been engineered for the local and sustained delivery of GEM and paclitaxel to PC, leading to a significant reduction in tumor growth [208]. This local delivery platform of integrin-targeted peptides on the surface of liposomal-like vesicles offers the advantage of achieving maximum therapeutic efficacy while minimizing toxic side effects to normal tissues. Additionally, an increasing number of molecules targeting integrins have been developed and investigated for molecular imaging in different cancers [209,210]. A ^68^Ga-radiolabeled peptide tracer targeting integrin αvβ6 has shown the ability of diagnostic imaging and post-surgery tumor relapse monitoring by performing preliminary clinical PET imaging on PC patients [211]. As for integrin β1, Li et al. developed a ^68^Ga-radiolabeled peptide tracer targeting the integrin α3β1 and unveiled significant radioactivity accumulation in PC cells and high tumor uptake in a xenografts mouse model [212]. With molecular imaging becoming an indispensable tool for noninvasive assessment of biological processes at the cellular or molecular level, we believe that integrin β1-based tracers will achieve high sensitivity and specificity when detecting lesions commonly missed by other technologies.

PTMs on integrins have been extensively studied in tumors due to their implications in tumor pathogenesis. Integrins can be regulated by many types of PTMs, including phosphorylation, glycosylation, ubiquitination, nitrosylation, and acetylation [213]. During these PTMs, glycosylation has been relatively well studied. Although integrin-mediated adhesion is based on the binding of α and β subunits to a defined peptide sequence, the strength of this binding and hetero-dimer formation can be modulated by various factors, including the status of *N*-glycosylation of integrin [214]. For example, integrin α5β1 contains 26 potential N-linked glycosylation sites (14 in the α subunit and 12 in the β subunit); previous studies from our group clearly showed that *N*-glycosylation on the β-propeller domain of the α5 [215] and/or the I-like domain of the β1 subunit [216] is essential for α5β1 expression on the cell surface. Moreover, remodeling of *N*-glycans on the integrin regulates its biological functions, including cell adhesion, migration, and proliferation [217,218,219,220,221,222,223,224]. In PC, Kuo et al. demonstrated that O-glycans on integrin β1 are modified by C1GALT1 and can regulate cell-ECM adhesion, which is associated with decreased tyrosine phosphorylation of FAK at Y397 in PC cells. Additionally, C1GALT1 also modifies O-glycans on α integrins, especially the αV and α5 subunits, and promotes PC cell invasiveness through the integrin–FAK signaling [225]. It is worth mentioning that deficiency of α1,6-Fucosyltransferase (FUT8), a sole enzyme responsible for catalyzing core fucosylation, inhibited cell migration and proliferation in both MIA PaCa-2 and PANC-1 cells, which may arise through the fucosylation on β1 integrin [226], suggesting FUT8 may be a potential therapeutic target for PDAC. Further studies are needed to focus on the role *N*-glycosylation of integrin β1 and its related glycosyltransferases during PC progression. Apart from glycosylation, phosphorylation, ubiquitination, nitrosylation, and acetylation are common PTM forms. In addition to other PTMs, tyrosine 792 in the membrane-proximal NPXY motif of the β integrin is selectively de-phosphorylated by density-enhanced phosphatase-1 (DEP-1), which further triggers integrin activation via talin recruitment and interferes with EGFR signaling [227]. Such roles of integrin β1 phosphorylation in PC have not been reported. Still, these findings suggest that strategies aimed to alter the PTM processes of integrin β1 are a promising approach to control PC progression.

## 7. Conclusions

Integrin β1, as the most common β subunit of the integrin family, has been confirmed to play a vital role in tumor initiation and progression and is responsible for regulating cell behaviors such as survival, proliferation, apoptosis, invasion, and migration differentiation, as well as CSC property. In this review, the examples given here highlight the intricate involvement of integrin β1 in every step of PC progression, from primary tumor formation to metastasis, indicating integrin β1 as an appealing target for the development of PC therapies. Although many preclinical efforts showed promising data, integrin β1-targeting PC therapies are few and often unsuccessful in clinical trials. These clinical failures reflect a tremendous challenge in translating the preclinical research on integrin β1 into clinically efficacious drugs. Further in-depth understanding of the molecular mechanisms regulating the heterogeneity and redundancies of integrin β1 functions will become the theoretical basis and guidance for developing appropriate clinical pharmacodynamic biomarkers to measure target engagement and proposing an alternative approach targeting integrin β1 in other cells contained in the TME. Additionally, nascent and developing strategies exploring integrin β1-based approaches as targets for the delivery of existing anticancer drugs and noninvasive tumor imaging will become a new research direction in the field of PC diagnosis and therapy.

## Figures and Tables

**Table 1:** Overview of cellular functions of reported β1 integrins in pancreatic cancer.

No.	Integrin	Year	Cell Lines	Expression	Functions												Mechanism	Model Used	Reference PMID
					Proliferation	Cell Cycle	Apoptosis	Angiogenesis	Adhesion	Migration	Invasion	CSC	Therapy Resistance	Tumor Growth	Tumor Metastasis	ECM Remodeling			
1	β1	2003	MIA PaCa-2, BxPC-3	up					+		+						GDNF/integrin β1	cell	12883269
2	β1	2005	SW1990, Capan-2	up					+		+				+		GDNF/integrin β1	cell	15999351
3	α6β1	2006	BxPC-3, Capan-2, SW1990	up	+				+	+							IL-1α/integrin β1 and uPA/uPAR/Ras, ERK	cell	16504015
4	β1	2006	Panc-1	up					+								CCK2/integrin β1/Src, PI3K	cell/animal	16547500
5	β1	2006	Panc-1, BxPC-3	up	+												integrin β1/FAK/B-catenin phosphorylation/-Lef/Tcf	cell	16651417
6	β1	2007	PATU8902, MIA PaCa-2, Panc-1	up									+				Cav-1/integrin β1/FAK	cell	17471232
7	β1	2007	Capan-1	up			+										p16INK4a/glycosylation/integrin β1 maturation	cell	17535296
8	β1	2008	BxPC-3, Panc-1, SW1990	up							+						integrin β1/ERK1/2 phosphorylation	cell	17688882
9	β1	2007	Panc-1	up					+		+						Np-1/integrin β1/FAK	cell	17726369
10	β1	2008	Capan-1, Colo-357, AsPC-1, BxPC-3, MIA PaCa-2, Panc-1	up	+				+	+							-	cell	18754866
11	β1	2008	HPDE6	up							+						CX3CR1/integrin β1/FAK	cell/animal	18974152
12	β1	2009	MIA PaCa-2	up					+	+	+							cell	19825166
13	β1	2010	bEnd.3, MEF	up				+						+			Fbln5/integrin β1/ROS	cell/animal	20197418
14	β1	2011	FG, Colo-357	up	+				+	+				+	+		-	cell/animal	21491421
15	β1	2011	Panc-1, PSN-1, MIA PaCa-2	up									+				PSC/integrin β1/FAK/radioresistance	cell/animal	21558392
16	β1	2011	Panc-1	up							+						FAP/ECM/integrin β1	cell	21668992
17	β1	2011	Panc-1, FG-Met2	up					+		+						-	cell	21678462
18	β1	2011	T3M4, BxPC-3, COLO-357	up	+					+	+		+				DNp63a/EGFR, integrin β1/drug resistance	cell	22053213
19	β1	2012	Panc-1, AsPC-1	up						+							integrin β1/Rho	cell	22232555
20	β1	2012	Panc-1	up								+					-	cell	22335271
21	β1	2012	PT45-P1	up						+	+						L1CAM/integrin β1/FAK/NF-κB/IL-1β/EMT	cell	22764136
22	β1	2013	Capan-2, FG, Colo-357, Panc-1, Panc1-MUC1	up	+					+	+			+	+		core 3 synthase/integrin β1/FAK	cell/animal	23754791
23	α2β1	2014	Panc-1, UlaPaCa	up					+	+							integrin α2β1/FAK	cell	24201748
24	β1	2014	Panc-1, AsPC-1,MIA PaCa-2	up					+	+	+				+		p53/Myo10/integrin β1/filopodia-inducing	cell/animal	24487586
25	β1	2014	AsPC1, BxPC-3, CFPAC-1, Panc-1, SW1990	up	+		+			+	+						integrin β1/FAK, AKT, and ERK/Gli-1/EMT	cell	24720337
26	β1	2014	MIA PaCa2, BXPC-3 ASPC-1, Panc-1	up													GD3/integrin β1/FAK/AKT	cell	24842107
27	β1	2014	PANC-1, MIA PaCa-2	up						+	+						eEF-2K/TG2/integrin β/Src/uPAR/MMP-2/EMT	cell	25215932
28	α2β1	2014	BxPc-3, Capan-1, Panc-1	up	+				+					+			integrin β1/FAK/ERK1/2	cell/animal	25336636
29	β1	2015	AsPC-1, Panc-1, MIA PaCa-2	up						+	+				+		EPAC1/PKC/integrin β1 trafficking and activation	cell/animal	25385424
30	β1	2015	AsPC-1, Capan-1, SU.86.86, PANC-1	up	+				+	+	+				+		-	cell/animal	25449434
31	β1	2016	ASPC-1, Panc-1, Suit-2	up						+	+						PHLPP/AKT/integrin β1	cell	26760962
32	β1	2016	PSC	up												+	integrin β1/ECM/matrix remodeling	cell	27170254
33	β1	2016	MIA PaCa-2, AsPC-1	up									+				integrin β1/Cdc42, AKT	cell	27289231
34	β1	2017	MIA PaCa-2, AsPC-1, BxPC-3, Panc-1, Capan-2,SW1990	up	+									+			REGF receptor, neuropilin-1/integrin β1/Src-AKT bypass signaling	cell/animal	27797376
35	β1	2017	Panc-1, L3.6pL, MIA PaCa2	up					+	+							NR4A1/p300/Sp/integrin β1	cell	28418095
36	β1	2017	AsPC-1, BxPC-3, CFPAC1, Panc-1	up											+		Fyn/P21-activated kinase 1/hnRNP E1/the alterative splicing of integrin β1.	cell/animal	28560430
37	β1	2017	BxPC-3, Capan-1, MIA PaCa-2	up	Y					+		+			+		integrin β1/FAK	cell/animal	28692661
38	α2β1	2018	Panc-1	up	+				+						+		integrin β1/JNK, ERK kinases, Src	cell/animal	28916526
39	β1	2017	AsPC-1, BxPC-3, Panc-1	up						+	+				+		integrin β1/EGFR/ERK/MAPK/EMT	cell/animal	29072694
40	β1	2018	PSC	up	+						+			+	+		GAL3/integrin β1/ILK/NF-kB/IL-8	cell/animal	29274868
41	β1	2018	MIA PaCa-2, Capan-1, AsPC-1	up	+				+	+	+			+	+		MUC4/integrin β1/FAK/ERK	cell/animal	29777904
42	β1	2018	MIA PaCa-2	up	+										+		VASP/integrin β1-FAK-YAP1/TAZ	cell/animal	29872721
43	β1	2018	Panc-1, SW1990, MIA Paca-2	up	+					+	+						miR-124/β1/phospho-FAK, phosphor-AKT, phospho-EEK1/2	cell	29988949
44	β1	2018	Panc-1	up			+										integrin β1/Cdc42	cell	30241340
45	β1	2018	AsPC-1	up									+				integrin β1/Cdc42/p110b/PI3K	cell/animal	30243721
46	β1	2018	Panc-1, PK59	up					+		+						H19/integrin β1,CD24	cell	30410672
47	β1	2019	Capan-1,BxPC-3	up	+					+		+					integrin β1/FAK/EMT	cell	30747824
48	α11β1	2019	myCAF	up						+						+	-	cell	31159419
49	α5β1	2019	MIA PaCa-2, SW1990, CFPAC-1, PANC-1, AsPC-1, BxPC-3, Panc 03.27	up						+					+		TGF-β/TFEB/RAB5A/α5β1 endocytosis	cell/animal	31387632
50	β1	2019	MIA PaCa-2	up	+												integrin β1/c-Myc degradation	cell	31452837
51	β1	2019	SW1990, AsPC-1, Panc-1, BxPC-3	up	+		+						+				miR-760/MOV10/integrin β1	cell	31693728
52	α3β1	2020	AsPC-1, MIA PaCa-2	up	+								+	+	+		ZIP4/ZEB/α3β1/JNK/ENT1/drug resistance	cell/animal	31711924
53	β1	2020	Panc-1, BxPC-3, MIA PaCa-2	up						+							HLA-B/integrin β1	cell	32194036
54	β1	2020	PANC-1	up						+	+						integrin β1 and Heparan Sulfate Dual-Targeting/YAP	cell	32266811
55	β1	2020	iKras*p53* PC cells	up									+				integrin β1/Kras	cell	32636409
56	β1	2020	Panc-1	up	+					+							integrin β1/FAK, AKT, ERK1/2, NF-κB	cell	33086527
57	β1	2022	Panc-1	up		+	+		+								FxOH/integrin β1/FAK, paxillin, FYN, AKT, PPARγ	cell	33590779
58	β1	2021	Panc-1	up	+												mi-16/integrin β1/PI3K/AKT	cell	33591944
59	β1	2021	Panc-1, MIA PaCa-2	up	+					+				+			hERG1/integrin β1 complex/AKT, HIF-1α	cell/animal	34045227
60	β1	2022	MIA PaCa-2	up									+				-	cell	34481933
61	β1	2021	MIA PaCa-2	up					+								integrin β1/kindlin-2/TGF-β receptor 2/Smad2/3	cell	34638957
62	β1	2022	adipose-derived mesenchymal stem cells	up	+					+				+			Mucin 5AC/CD44-integrin β1/Rac1	cell/animal	35219699
63	β1	2021	CF Pac-1, SW1990	up						+							RAB5A/integrin β1/Cdc42	cell	33341673

“+” means that the integrin plays a role in related cancer cell biological functions. * iKras*p53* PC means PC cells derived from transgenic mice “iKras*p53* mice”.

**Table 2:** Overview of the clinical significance of integrin β1 in pancreatic cancer.

No	Year	Detection Method	Source	Expression	No. of Patients	Lymphatic Invasion	Distance Metastasis	TNM Stage	AUC ^a^	Survival	Prognostic Marker	Reference PMID
1	2012	PCR	PBMC ^b^	up	37	+	+	+				22382453
2	2012	PCR	PBMC	up	30		+	+				22695923
3	2013	PCR, ELISA	PBMC, plasma	up	54	+	+	+		DFS ^c^	+	24004467
4	2016	IHC	tissue	up	63					OS ^d^, DFS	+	27289231
5	2017	IHC	tissue	up	68			+		OS	+	29072694
6	2018	IHC	tissue	up	30					OS, DFS	+	29988949
7	2019	IHC	tissue	up	102						+ (combined with PODXL/BCL7B)	31166991
8	2020	IHC	tissue	up	93							31711924
9	2021	public database	tissue	up	178				0.8635	OS, DFS	+	33591944

^a^. AUC: area under the curve; ^b^. PBMC: peripheral blood mononuclear cell; ^c^. DFS: disease-free survival; ^d^. OS: overall survival. “+” means that integrin β1 could be used as a prognostic biomarker in PC.
